# Left Bundle Branch Block: A Reversible Pernicious Effect of Lacosamide

**DOI:** 10.7759/cureus.10234

**Published:** 2020-09-03

**Authors:** Monil M Majmundar, Tikal Kansara, Palak Shah, Harshvardhan Zala, Shobhana Chaudhari

**Affiliations:** 1 Department of Internal Medicine, New York Medical College, Metropolitan Hospital Center, New York, USA; 2 Department of Internal Medicine, Smt. B.K. Shah Medical Institute and Research Center, Sumandeep Vidyapeeth, Vadodara, IND; 3 Department of Internal Medicine, Amidhara Hospital, Surat, IND

**Keywords:** left bundle branch block, lacosamide, av block, the sinus node, seizure

## Abstract

A 95-year-old male with a medical history of focal epilepsy presented with transient ischemic attack (TIA)/pre-syncope like symptoms. He was on lacosamide (LCM) and levetiracetam. On evaluation, he was found to have left bundle branch block (LBBB), sinus pause of three seconds, and 1st degree atrioventricular (AV) block. After holding LCM, electrocardiogram changes were reversed to baseline (before commencing LCM). In conclusion, to the best of our knowledge, this is the first case of reversible LBBB along with sinoatrial (SA) node and AV node dysfunction in an elderly male on LCM therapy.

## Introduction

Lacosamide (LCM) is a novel antiepileptic medication, approved in 2008, as adjunctive therapy for focal and secondarily generalized seizures. LCM acts by enhancing the slow inactivation of voltage-gated sodium channels. The drug has shown to provoke cardiac conduction abnormalities in the past. It has been associated with syncope, chest pain, atrioventricular (AV) blocks, atrial fibrillation, atrial flutter, and arrhythmias, including bradycardia. Other common side effects associated with LCM include diplopia, blurred vision, nausea, vomiting, headache, dizziness, and ataxia. No previous studies have shown a co-relation of LCM with left bundle branch block (LBBB) or sinus node dysfunction. Here, we report the first case of reversible LBBB along with sinoatrial (SA) node and AV node dysfunction in an elderly male on LCM therapy. Our aim is to make clinicians aware of reversible LBBB as a side effect with LCM.

## Case presentation

A 95-year-old Hispanic male presented to the emergency department (ED) with the development of sudden onset of slurring of speech, dizziness, and difficulty in getting up from the sitting position, which resolved en-route to the hospital. He endorsed a similar episode two days ago. He denied any loss of consciousness, confusion, motor or sensory deficits, blurring of vision, diplopia, chest pain, palpitation, syncope, and dyspnea during the episode. In the emergency room, his blood pressure was 190/72 mmHg, and his pulse rate was 80 per minute. Cardiovascular examination showed normal heart sounds, did not show any murmur or added sounds, carotid pulse was regular, with good volume and pressure. Neurological examination did not reveal focal deficits or any other abnormalities; examination of other systems was trivial.

The patient's medical history included hypertension and focal epilepsy disorder. His home medications were lacosamide (LCM) 200 mg twice a day, levetiracetam 1500 mg twice a day, lisinopril 40 mg once a day, and amlodipine 10 mg daily.

**Figure 1:**
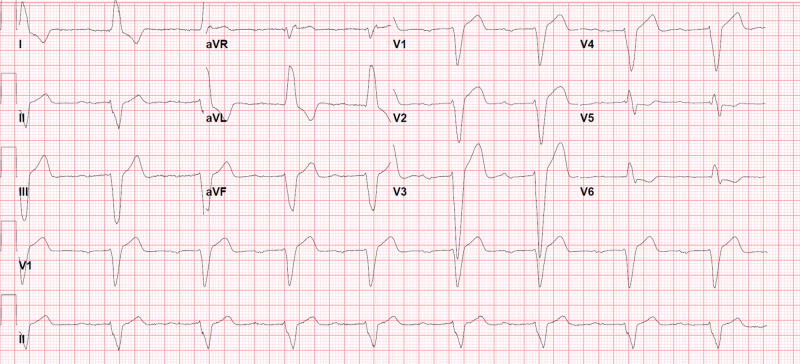
Electrocardiogram (EKG) on admission in January 2019 1st degree atrioventricular (AV) block, left bundle branch block (LBBB), PR interval 378 msec, QRS 200 msec

Fingerstick, complete blood count, basic metabolic profile, hepatic function, urine toxicology, were nugatory. Thyroid stimulating hormone (TSH), Troponin T, and serum magnesium level were within the normal reference range. Initial electrocardiogram (EKG) revealed normal sinus rhythm, 1st degree AV block, and LBBB (Figure 1). Computed tomography (CT) of the head with angiography, echocardiography, and carotid duplex were normal. 

**Figure 2:**
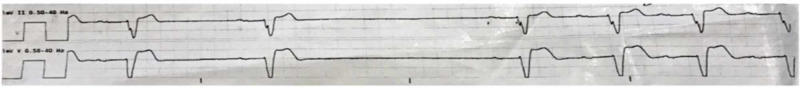
Telemetry recording Sinus pause of 3 seconds with a heart rate of 30 per minute

**Figure 3:**
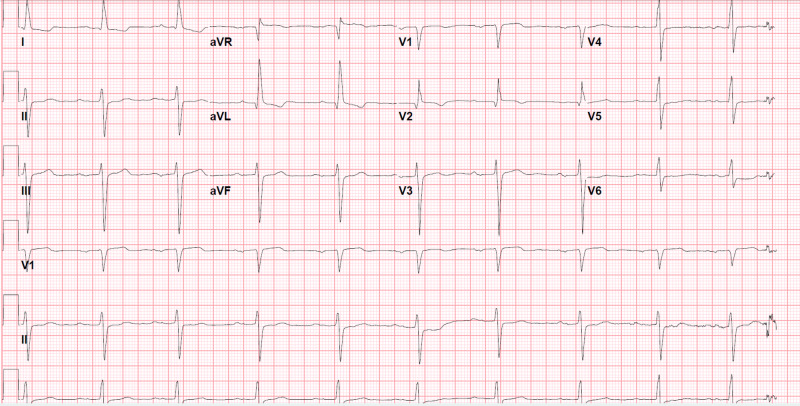
Electrocardiogram (EKG) 24 hours after holding lacosamide Resolution of left bundle branch block (LBBB), 1st degree atrioventricular (AV) block, PR interval decreased to baseline 270 msec, QRS 110 msec

**Figure 4:**
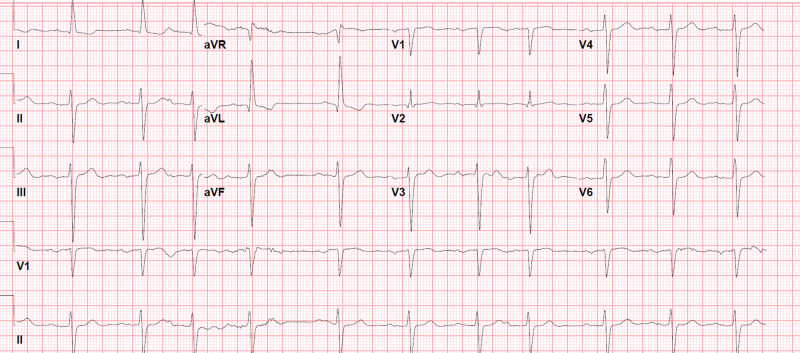
Electrocardiogram (EKG) before starting lacosamide in September 2016 1st degree atrioventricular (AV) block, PR interval 290 msec, QRS duration 112 msec

**Figure 5:**
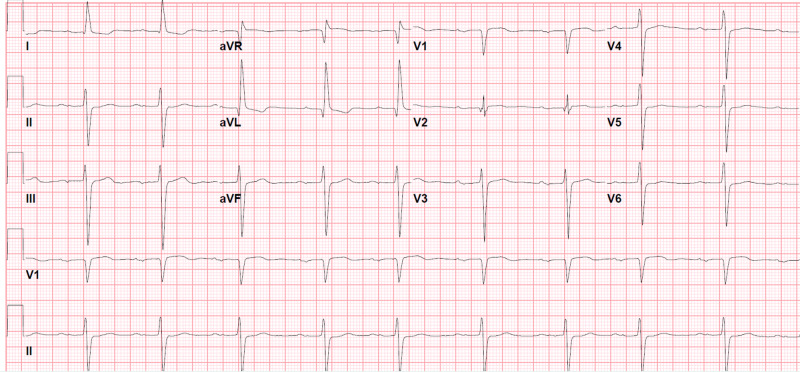
Electrocardiogram (EKG) on lacosamide 200 mg per day in September 2017 1st degree atrioventricular (AV) block, PR interval 322 msec, and QRS duration 134 msec

**Figure 6:**
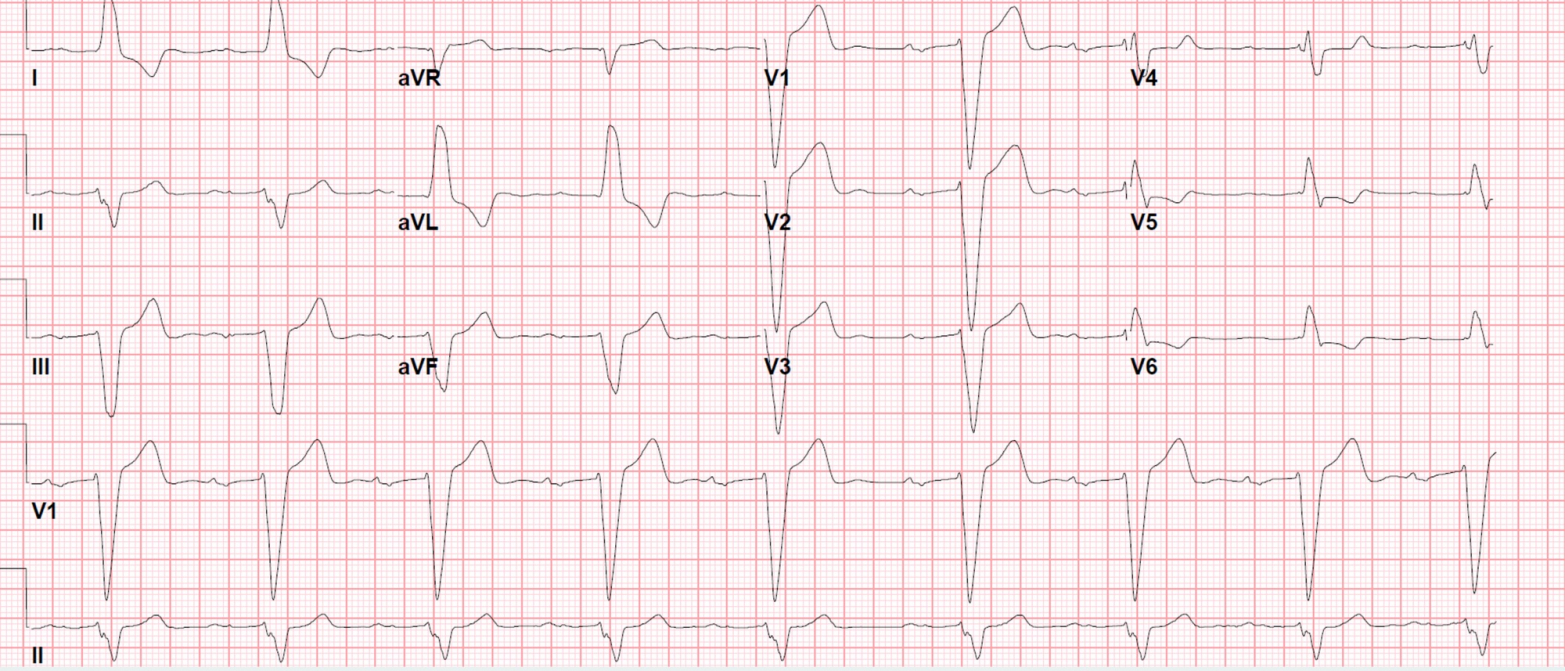
Electrocardiogram (EKG) on lacosamide 400 mg per day in September 2018 left bundle branch block (LBBB), 1st degree atrioventricular (AV) block, PR interval 378 msec, QRS duration 200 msec

**Figure 7:**
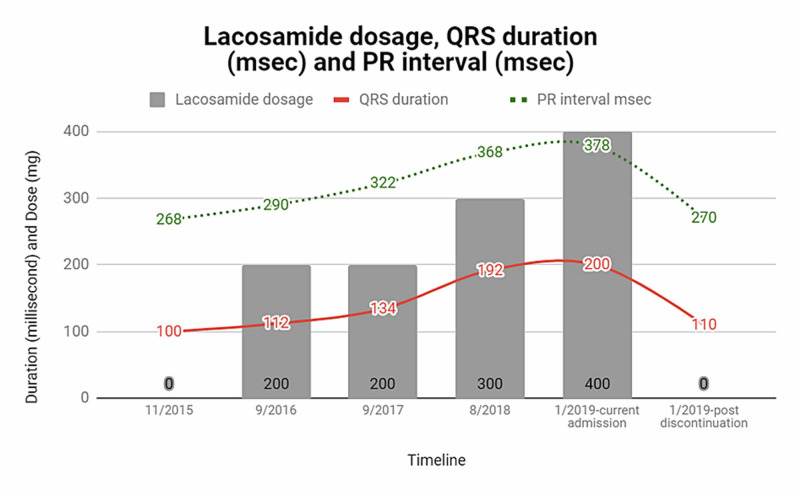
Graphical representation of lacosamide (LCM) dose and PR interval, QRS duration. Baseline PR, QRS; With the increase in LCM dose, PR interval, and QRS duration increases; PR, QRS back to baseline after holding.

The patient was admitted to the telemetry unit. Telemetry captured intermittent sinus bradycardia with heart rate as low as 30/min and SA node arrest of 3 seconds (Figure 2). The serum LCM level was sent. His LCM was switched over to topiramate and was observed in the coronary care unit (CCU) where he received an external pacemaker. Twenty-four hours after withdrawal of LCM, the patient’s EKG (Figure 3) did not divulge any sinoatrial node arrest; heart rate returned to a baseline of 54 per minutes; LBBB obliviated with QRS of 110 milliseconds (msec), and a result of LCM serum level was 15.8 mcg/ml (elevated). Subsequently, the patient was discharged post-24-hour observation. On review of records, we correlated the timeline from the initiation of LCM, its dose increment, and corresponding EKG readings (Figure 4-6). Each figure shows the dose of LCM and the corresponding increase in PR interval and QRS complex duration. The dose of LCM was increased for optimal seizure control. Figure 7 demonstrates the graphical representation of the correlation between LCM dosage, PR interval, and QRS interval.

The patient was followed up in the clinic within a month of discharge. His repeat EKG in the clinic was normal. A follow-up with the Cardiology clinic in the next six months found the patient to have sick sinus syndrome and a permanent pacemaker was placed. The patient has been asymptomatic since then on routine follow-up. 

## Discussion

LCM is known to be associated with several adverse cardiac events. LCM, at the recommended dose, can induce dose-dependent PR interval prolongation [1,2,3] and first-degree AV block [1,3,4]. LCM at a higher dose (600 mg/day) produces atrial flutter/fibrillation [1,5], sinus node dysfunction (500 mg/day) [6] and second/third-degree AV block [7,8]. Two clinical studies reported fatal cardiac arrests [1,2].

Here, we report an association between LCM and LBBB characterized by prolonged QRS duration, SA node dysfunction indicated by sinus pause, and AV block indicated by prolonged PR interval. This patient’s baseline (in September 2016) PR interval and QRS duration were 290 msec and 112 msec, respectively (Figure 4). After the introduction of LCM with 200 mg per day in September 2016 due to the development of focal seizure, PR interval and QRS duration increased to 322 msec and 134 msec (EKG from September 2017) (Figure 5). The patient had another episode of seizure, following which the neurologist had augmented the dose to 300 mg per day in September 2017 and subsequently 400 mg per day in March 2018, and the patient developed LBBB with increment in PR interval to 378 msec and QRS duration to 200 msec (in September 2018) (Figure 6) which went unnoticed. After the admission to the telemetry floor in January 2019, telemetry recorded a sinus pause of 3 seconds (Figure 2) which might have led to episodes of pre-syncope. Elevated serum LCM level and resolution of EKG changes after holding LCM support LCM as a culprit of the patient’s clinical presentation and all EKG findings. Seizures can have a detrimental effect on the heart by causing tachycardia, syncope, hypoxia, or even sudden unexpected death (SUED).

In vitro studies have shown that LCM inhibits repetitive neuronal firing and stabilizes the neuronal membrane by enhancing the slow inactivation of sodium channels. There is no effect on fast inactivation of sodium channels. Clinically, PR and QRS prolongation are results of the AV nodal and infra-hisian conduction delay which generates action potentials (AP) through voltage-gated sodium channels. LCM’s inhibition of sodium channels can explain the hypothesis of PR and QRS prolongation. The development of LBBB with LCM can be explained only if LCM acts on calcium channels. However, none of the literature mentions the action of LCM on calcium channels, and hence its effect of sinus node dysfunction demands further research. 

## Conclusions

To the best of our knowledge, this is the first reported case of reversible LBBB associated with LCM. A dose-dependent increase in PR interval and QRS duration was found with sequential increase in LCM dose which was completely reversed by discontinuation of the drug. This case emphasizes the need for routine monitoring of EKG and LCM levels during every dose increment and at regular intervals. Based on the available data, close monitoring is required for patients taking LCM with underlying conduction abnormalities.
